# A comment on the connection between BA10 and episodic memory

**DOI:** 10.3389/fnbeh.2023.1105168

**Published:** 2023-05-05

**Authors:** Yifat Faran

**Affiliations:** School of Social Work, Ashkelon College, Ashkelon, Israel

**Keywords:** Brodmann area 10, episodic memory, posterior hippocampus, knowledge integration, retrieval

## Introduction

This article is a commentary on the role of BA10 in episodic memory, as predicted by Ben Shalom and Bonneh's (2019) model of the narrow prefrontal cortex. It aimed to explore whether there is any existing literature on memory that supports a connection between BA10 and episodic memory, and if so, what form this connection might take.

## Historical context

Many studies have emphasized the crucial role of the frontal lobes in episodic memory (e.g., Piolino et al., 2007; Coste et al., 2015; for a review, see Vakil, 2023). Based on lesion studies, Stuss and Alexander (2005) suggested that the frontal lobes are involved in multiple strategic processes. Similarly, Moscovitch (1992) suggested that the frontal lobes support the memory system by applying top-down processes, such as the implementation of strategy, organization, and conceptual elaborative encoding and retrieval. However, as Stuss and Alexander contended, the frontal lobe is not a homogenous structure and has to be considered in view of its component parts. The most efficient subdivision is based on histology and is defined by Brodmann areas. This article focused on the prefrontal pole, known as Brodmann area 10 (BA10). Studies have shown the involvement of BA10 in many cognitive tasks, including prospective memory (Burgess et al., 2007; Raskin et al., 2018), planning (Volle et al., 2011), analogy solving (Qiu et al., 2008), multitasking (Gilbert et al., 2007; Roca et al., 2011), and more (for a review, see Snow, 2016).

The current article examined the involvement of BA10 in episodic memory, specifically, the predictions made by Ben Shalom and Bonneh (2019) (i.e., that BA10 is involved in the integration of memory episodes) and by Ben Shalom (2009) (i.e., that medial BA10 is involved in the representation of memory episodes themselves).

On the anatomical level, BA10 shows anatomical connections with brain structures involved in episodic memory. Moayedi et al. (2015) found that BA10 can be divided into two sub-regions: the medial cluster and bilateral lateral clusters. The medial cluster is functionally connected to the bilateral and medial PFC, bilateral precuneus/posterior cingulate cortex, ipsilateral lateral occipital cortex, bilateral parahippocampal gyri, bilateral subgenual cingulate cortex, and bilateral middle temporal gyrus, which are mostly associated with the default mode network (DMN; e.g., Buckner and Krienen, 2013; Mak et al., 2017). The bilateral lateral clusters are connected to the bilateral supplementary motor area, ventrolateral premotor cortex, lateral parietal area, dorsolateral prefrontal cortex, and bilateral anterior insula, which are mostly associated with the central-executive network (CEN; Li et al., 2021). BA10 also shows functional connectivity during memory tasks. For instance, Fritch et al. (2021) found that BA10 was functionally connected to the posterior hippocampus, associated with retrieval, but not with the anterior hippocampus, associated with encoding. This functional connectivity was found during retrieval but not during encoding.

On the functional level, a growing body of substantial evidence supports the involvement of BA10 in episodic memory. Numerous studies have demonstrated the involvement of BA10 in episodic retrieval and, to a lesser extent, in episodic encoding. For example, Lepage et al. (2000) reviewed imaging studies that focused on episodic memory retrieval and found that many of them showed activation in BA10 (e.g., Schacter et al., 1996; Rugg et al., 1998). Since then, a growing body of evidence has supported the role of BA10 in the retrieval of episodic memory, and retrieval efforts. However, studies that focus on encoding found less activation in this region. For instance, Fletcher and Henson (2001) reviewed studies that used imaging to test brain activation during both encoding and retrieval and found that, while only 2 out of 23 studies showed activation in BA10 during encoding, 15 out of 25 studies showed activation in BA10 during retrieval. This region has therefore been labeled as part of the retrieval success network. In a review of research that tested activation in response to repetition, Kim (2017) found that BA10, as part of the retrieval success network, indeed showed increased activation due to repetition.

Similarly, Weymar et al. (2018) reported that repetition enhancement was found in the medial posterior parietal (precuneus/cuneus), lateral parietal cortex (angular gyrus), and left BA10. However, some findings were less consistent with the idea that BA10 is involved in the integration of memory episodes. For example, King et al. (2005) showed that increasing the diversity between the contexts of the events, such as giving each item a different context to make them more distinct, reduced the activation in BA10.

## A recent synthesis

Two questions can thus be asked regarding the connection between BA10 and episodic memory. The first question pertains to why BA10 is more active during retrieval than encoding. The second question concerns the nature of the actual connection between BA10 and episodic memory.

Some answers to both questions might lie in a recent study by Bonasia et al. (2018). In this article, the authors tested brain activation during the encoding and retrieval of video clips. Participants saw video clips that were either similar to events people encounter in everyday life, that is, congruent video clips, or video clips that were very unusual and/or dissimilar to anything people encounter in day-to-day life, that is, incongruent video clips. In addition, the authors also used neutral video clips that were neither very similar nor dissimilar to everyday life. As expected, the participants recalled both congruent and incongruent video clips better than neutral ones, indicating that both congruency and incongruity can enhance memory. However, brain activation in medial BA10 during encoding and retrieval was modulated by congruency alone. In a parametric analysis, during encoding, medial BA10 was more activated with increasing congruency. It also showed more functional connectivity during encoding with increasing congruency. Importantly, during retrieval, medial BA10 also showed increased functional connectivity with the increasing congruence of the retrieved material. These findings are consistent with those of other studies that showed increased activation of BA10 during repetition (Kim, 2017; Weymar et al., 2018) and reduced activation when the context between encoding and retrieval was changed (King et al., 2005).

It thus appears that the answer to the first question, i.e., why is BA10 activated more during retrieval than during encoding?, might lie in the fact that studied items are rarely considered in terms of their level of congruency, rather, they are more commonly compared between retrieval and encoding. Thus, when the relevant factor is not the level of congruency but retrieval vs. encoding, retrieved items, which have already been encountered, are, on average, more congruent with prior context than encoded items, resulting in additional BA10 activation. In addition, regarding the second question, what does this synthesis mean for the connection between BA10 and episodic memory? According to Bonasia et al. (2018), the medial BA10 detects congruence between current experiences and prior knowledge before activating relevant prior knowledge to facilitate comprehension and enhance the integration of new event-specific information with prior knowledge.

## Discussion

As noted by Bonasia et al. (2018), their synthesis is consistent with Van Kesteren et al.'s (2012) SLIMM model (schema-linked interactions between medial prefrontal and medial temporal regions), according to which event congruence would affect activity and connectivity across the brain during both encoding and retrieval: increased congruence between events and prior knowledge correlating with activity in the medial prefrontal cortex, and increased incongruence between events and prior knowledge correlating with activity in the medial temporal lobe.

More importantly, the connection between BA10 and episodic memory indicates that incoming memory episodes are not represented in medial BA10. Instead, what is represented in medial BA10 is prior knowledge that, when activated, helps the integration of incoming episodes into prior knowledge. Thus, while there is indeed a connection between BA10 and episodic memory, as the Ben Shalom and Bonneh (2019) model predicted, it is not as straightforward as incoming memory episodes represented in medial BA10.

## Author contributions

The author confirms being the sole contributor of this work and has approved it for publication.
